# Digital coordination in mass casualty incidents: a retrospective analysis of prehospital distribution times before and after IVENA-MANV implementation

**DOI:** 10.1007/s00068-026-03216-2

**Published:** 2026-05-20

**Authors:** Vesta Brauckmann, Bastian Ringe, Marta Caviglia

**Affiliations:** 1 Hannover Medical School, Department of Trauma Surgery, Carl-Neuberg-Straße 1, D-30625 Hannover, Germany; 2 Hannover Medical School, Interdisciplinary Department of Emergency and Disaster Medicine, Carl-Neuberg-Straße 1, 30625 Hannover, Germany; 3 CRIMEDIM—Center for Research and Training in Disaster Medicine, Humanitarian Aid, and Global Health, Università del Piemonte Orientale, Novara, 28100 Italy; 4 Department of Translational Medicine, Università del Piemonte Orientale, Novara, Italy

**Keywords:** MCI, Prehospital times, Emergency medical services, Dispatch, Mass casualty

## Abstract

**Background:**

Digital coordination tools have been proposed to improve management during mass casualty incidents (MCIs). In 2021, the IVENA-MANV system was introduced in the Hannover region as an extension of the established IVENA eHealth platform to enable digital patient tracking and hospital capacity management. However, no real-world evaluations have yet quantified their operational impact.

**Objectives:**

This study aimed to provide the first real-world evaluation of the IVENA-MANV digital coordination system and to determine how its implementation affected prehospital distribution processes and time intervals during mass casualty incidents.

**Methods:**

A retrospective observational study to assess the impact of the IVENA-MANV digital coordination platform on prehospital times during MCIs in Germany was conducted using dispatch records and IVENA-MANV logs from all MCIs between January 2018 and July 2025 in the Hanover-Region in Germany. Primary outcomes were the triage-to-evacuation (TtE) and total prehospital time (TPHT) per patient. Mann-Whitney U tests compared pre- and post-implementation groups, and multiple linear regression models examined associations between IVENA-MANV use, MCI-level, and time intervals.

**Results:**

Seventy-three MCIs met inclusion criteria, including 188 documented individual casualty characteristics. 41.1% of incidents used IVENA-MANV. Most MCIs were trauma-related (65.8%) and traffic-associated (64.4%). Mean TtE was 40.2 ± 19.3 min, and mean TPHT 83.9 ± 29.5 min. No statistically significant differences were found between pre- and post-implementation cohorts (*p* > 0.05). Regression analysis confirmed MCI-level as the only significant predictor of prolonged intervals (*p* < 0.001). IVENA-MANV enabled structured hospital allocation and real-time capacity confirmation (mean 1.8 min to first confirmation) without extending on-scene times.

**Conclusions:**

Implementation of IVENA-MANV did not significantly affect prehospital time intervals but demonstrated qualitative benefits for coordination, documentation, and hospital communication. The value of digital systems in disaster medicine may therefore lie in strengthening coordination and information resilience rather than accelerating evacuation. Prospective multicentre and mixed-methods studies are warranted to assess their broader system-level impact.

**Supplementary Information:**

The online version contains supplementary material available at 10.1007/s00068-026-03216-2.

## Introduction

Mass casualty incidents (MCIs) represent one of the most complex challenges to prehospital emergency services [1, 2]. They are defined by a sudden and significant mismatch between the number of casualties and the available medical resources within a given region, often resulting in severely strained emergency response capacities [3, 4]. These events demand coordinated allocation of limited resources under time pressure, compounded by uncertainty, communication breakdowns, and logistical challenges, often within a chaotic and rapidly evolving environment [2, 3, 5–7]. 

Among the key components of successful MCI management is the optimization of prehospital time intervals [8, 9]. The total prehospital time, defined as the time from initial MCI activation to patient handover at a hospital, has been highlighted as determinant of patient outcome in critically injured patients [7, 10, 11]. Efficient evacuation also serves to decompress the disaster scene, enabling better use of field resources and timely access to definitive care for critically injured patients [4, 8, 9]. 

Despite significant progress in triage systems, evacuation protocols, and treatment algorithms, coordination difficulties remain a recurring issue in the prehospital phase of MCIs [7]. Inadequate information flow and lack of situational awareness can lead to resource misallocation, uneven hospital loads, and treatment delays [6]. In response, digital coordination tools have emerged to address these issues [2, 8, 12, 13]. Simulation-based studies, such as the SOGRO model, have demonstrated the theoretical potential of digital systems to improve distributed decision-making and interoperability in German disaster scenarios [14, 15]. However, they have not yet been implemented in real operations.

In this context, the IVENA eHealth MANV (Interdisziplinärer Versorgungsnachweis für das Massenanfallgeschehen, engl.: “Interdisciplinary Care Capacity Registry for Mass Casualty Incidents”), hereafter referred to IVENA or IVENA-MANV, system was launched in 2021 and introduced in the EMS in the region of Lower Saxony, Germany [16, 17]. As a digital coordination platform, it aims to support hospital assignment based on triage category and real-time capacity. The system is integrated with mobile devices and barcode-based patient identification and is designed to provide live access to regional hospital capacities for dispatch centers, emergency physicians, and hospital personnel. A preliminary evaluation of IVENA-MANV suggested potential benefits for communication and resource allocation [16], yet, no quantitative data on operational or time-based performance metrics have been published to date. The absence of quantitative outcome is further reflected in a recent systematic review of MCI management strategies, which included no real-world evaluations of digital coordination tools, an omission that underscores the current lack of empirical evidence in this area [7]. 

This study addresses this gap by evaluating the impact of the IVENA-MANV digital coordination system on prehospital time intervals during mass-casualty incidents. By providing real-time visibility of hospital capacities and enabling digital allocation of patients by triage category, IVENA-MANV has the potential to streamline coordination between the scene, dispatch centers, and receiving hospitals, thereby improving the timeliness of patient evacuation. To test this mechanism, the study focuses on quantitative operational outcomes, specifically prehospital time intervals, before and after system implementation. By providing empirical data on digital coordination outcomes in the German prehospital setting, this research aims to contribute to evidence-based optimization of disaster response systems.

## Methods

### Study design and population

We conducted a retrospective observational study to evaluate the impact of the IVENA-MANV digital coordination platform on prehospital time intervals during MCIs. The study area encompassed the Hannover region of Lower Saxony, Germany, under the jurisdiction of the Regional Fire Brigade Dispatch Center. All officially registered events occurring between January 30, 2018, and July 31, 2025, were eligible for inclusion. We stratified the data into two distinct study periods: the pre-implementation phase, spanning from January 30, 2018, to December 31, 2020, and the post-implementation phase, from January 1, 2021, to July 31, 2025. Incidents were excluded if the alarm did not result in patient transport or if core operational timestamps were absent. The primary unit of analysis was the individual patient transport (T_n_), with the incident serving as the clustering unit for descriptive and inferential purposes. We further excluded individual patient transports if a hospital arrival timestamp was missing in the dispatch records, as this was the requisite for verifying completed evacuations. To ensure regional consistency and reduce selection bias, we screened all documented MCIs within the 7.5-year study period according to predefined data completeness criteria.

### Data collection and timestamp validation

We extracted and cross-referenced data from two primary sources: the COBRA dispatch management system (Vappweb Cobra-Einsatzrecherche) and the IVENA-MANV logs. COBRA serves as the primary operational database for the Hannover dispatch center, logging all vehicle movements and radio communications. All primary outcome variables, including triage-to-evacuation (TtE), total on-scene time (TOS), and total prehospital time per patient (TPHT), were derived exclusively from COBRA radio-status updates. These updates are manually triggered by ambulance crews via standardized radio buttons (Status 3—departure; 4—arrival at scene; 7—departure from scene; 8—arrival at hospital). Secondary timestamps specific to digital coordination, such as hospital capacity confirmations and allocation decisions, were extracted from IVENA-MANV logs to characterize the digital workflow. We noted that triage documentation timings were recorded at the incident level; in the pre-implementation period, these were manually entered into COBRA following verbal radio communication, whereas in the post-implementation period, they were digitally logged within IVENA-MANV.

### Operational definitions and descriptive variables

**Fig. 1:**
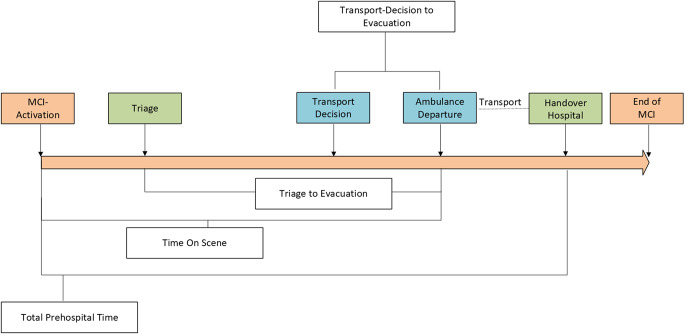
Distribution of prehospital time-intervals in a mass casualty incident. The intervals are defined as following: Total Prehospital Time: interval from MCI activation to hospital handover; Time on scene: interval from MCI activation to ambulance departure; Triage to Evacuation: interval from first triage to ambulance departure; Transport decision to evacuation: Interval from logged hospital allocation decision to ambulance departure

We defined the primary outcomes as TtE (first triage to ambulance departure), TOS (activation to departure), and TPHT (activation to hospital arrival). The relationship and definitions of these intervals are illustrated in Fig. 1. To provide a comprehensive profile of each incident, we recorded several descriptive variables. MCI characteristics included the date, time of day, and the specific type of event, categorized into traffic accidents, fires/explosions, intentional violence, or CBRN hazards. The clinical profile was further classified as primarily trauma, medical/toxicological, or a combined manifestation. MCI severity levels were defined according to regional classification: MANV 5 (up to 5 casualties), MANV 10 (up to 10), MANV 20 [11–20], and higher levels (50, 100, 200) signifying catastrophic scales. Casualty characteristics included the total number of patients and their assigned triage category (Red/Immediate Priority; Yellow/Urgent; Green/Minor Injuries; Blue/Unsalvageable; Black/Deceased). Where available, we also documented age, suspected diagnoses, and trauma mechanisms. Furthermore, we recorded the modes of transport, distinguishing between ambulances, Emergency Physician Vehicles (EPV), non-emergency ambulances, and Helicopter Emergency Medical Services (HEMS). To assess the balance of patient distribution, we calculated the coefficient of variation (CV) for incidents involving transports to two or more hospital types. The CV was defined as the ratio of the standard deviation (SD) to the mean number of patients per hospital type (µ) expressed as percentage (CV = [SD/ µ] x 100). Hospital allocation appropriateness for the post-implementation cohort was evaluated by cross-referencing triage priority with facility capability. According to regional protocols, appropriateness required Red patients to be transported to Level I or II trauma centers and Yellow patients to Level I–III centers. Allocations to pediatric hospitals or transports involving documented non-trauma diagnoses were considered appropriate regardless of the triage category.

### Triage protocols and traditional workflow

In both study groups, first responders perform primary triage using a structured xABCDE-format and mSTaRT-based checklist [18, 19], assigning categories via color-coded sliders. Secondary triage is conducted by emergency physicians to adjust categories, assign transport priority, and define diagnoses for clinical matching. Before the introduction of IVENA-MANV, MCI coordination relied on a mostly analogue, fragmented workflow. Hospital assignment was performed by the dispatch center and leading emergency physician using predefined allocation matrices supplemented by radio and telephone communication. Triage documentation was paper-based, and patient assignment occurred sequentially without a shared real-time overview of system-wide capacity. While a basic version of IVENA allowed individual ambulance registration, it lacked MCI-specific identifiers and was not used during MCIs. Incident management was handled entirely through COBRA and verbal communication, with the dispatch center manually entering radio-transmitted triage information into the COBRA system.

### The IVENA-MANV Intervention

The IVENA-MANV system, operational since 2021 as an extension of the regionally established IVENA-eHealth infrastructure, provides dispatch centers and hospitals with a shared, continuously updated overview of treatment capacities and specialty availability. It specifically supports real-time triage documentation, patient tracking, and dynamic hospital assignment during MCIs. The technical implementation required integration with dispatch systems, hospital emergency department terminals, and mobile data devices. The system utilizes a patient assignment code (PZC) encoding triage category and presumptive diagnosis. When a patient is registered via the mobile interface, receiving hospitals are automatically notified of triage status, transport priority, and estimated time of arrival (ETA). During an MCI, patients are tagged with a wristband carrying a unique identifier, QR-code, and RFID-chip for analog-digital interfacing. The application provides real-time capacity data, dynamic filtered patient lists, and digital allocation. If digital access is unavailable, documentation reverts to an analog barcode-based backup. These operational workflows are consistent with regional Disaster Medical Standard Operating Procedures [16, 17]. 

### Statistical analysis

The analysis was performed using SPSS Statistics 29.0 (SPSS Inc., IBM, Chicago, IL, USA). Descriptive statistics characterized incident and casualty profiles. Due to the non-parametric distribution of time variables, we conducted group comparisons using Mann-Whitney U tests. To account for the evacuation progression at the transport level, we stratified the analysis chronologically for each individual transport sequence (T_1_ through T_9_). We constructed multiple linear regression models with time intervals as dependent variables and IVENA-MANV implementation and MCI level as predictors. To account for the non-independence of individual patient units within the same incident, these models utilized cluster-robust standard errors. Significance was defined as *p* < 0.05. Finally, we performed a post-hoc power analysis to determine effect sizes and statistical power.

## Results

Between 30 January 2018 and 31 July 2025, a total of 2 429 034 dispatches were recorded in the Hannover region. After excluding non-relevant alarms and records with incomplete data, 73 MCIs with at least two patient transports were included. Unless otherwise specified, all subsequent time interval analyses were performed at the level of individual patient transport, with incidents used as clustering units for descriptive purposes. Of these, 30 MCIs occurred after the implementation of IVENA-MANV.Table 1 summarizes MCI characteristics before and after IVENA-MANV implementation. Across the 73 MCIs, the majority were level 5 or 10 incidents (94.5%), predominantly trauma-related and traffic accidents. MCIs occurred mostly in urban areas (47.9%) and were evenly distributed across seasons and weekdays, with a slight summer peak and more events from Monday to Wednesday. Time-of-day distribution showed a modest concentration in the afternoon (12:01–18:00, 35.6%). Although the post-implementation cohort contained proportionally more MCI-10 incidents and fewer high-level MCIs, these differences were modest and did not meaningfully affect the overall comparability of the groups in terms of incident type, clinical category, or localization.

**Table 1:** MCI-Characteristics

	All MCIs n (% *N* = 73)	Before IVENA-MANV n (% *N* = 43)	After IVENA-MANV n (% *N* = 30)
**MCI-Level**			
MCI 5	37 (50.7%)	26 (60.5%)	11 (36.7%)
MCI 10	32 (43.8%)	15 (34.9%)	17 (56.7%)
MCI 20	2 (2.7%)	2 (4.7%)	0
MCI 25	2 (2.7%)	2 (4.7%)	0
**Change of MCI-Level**	4 (5.5%)	2 (4.7%)	2 (6.7%)
**Type of MCI**			
Traffic Accident	47 (64.4%)	27 (62.8%)	20 (66.7%)
Fire	23 (31.5%)	15 (34.9%)	8 (26.7%)
CBRN	2 (2.7%)	0	2 (6.7%)
Other	1 (1.4%)	1 (2.3%)	0
**Clinical Category of MCI**			
Trauma-MCI	48 (65.8%)	28 (65.1%)	20 (66.7%)
Medical-MCI	3 (4.1%)	1 (2.3%)	2 (6.7%)
Combined Trauma/Medical-MCI	22 (30.1%)	14 (32.6%)	8 (26.7%)
**Localization of MCI**			
Highway	20 (27.4%)	10 (23.3%)	10 (33.3%)
Rural Road	18 (24.7%)	12 (28.9%)	6 (20.0%)
Urban area	35 (47.9%)	21 (48.8%)	14 (46.7%)
**Season**			
March-May	15 (20.5%)	10 (23.3%)	5 (16.7%)
June-August	26 (35.6%)	13 (30.2%)	13 (43.3%)
September-November	21 (28.8%)	14 (32.6%)	7 (23.3%)
December-February	11 (15.1%)	6 (14.0%)	5 (16.7%)
**Day of the week**			
Monday-Wednesday	37 (50.7%)	19 (44.2%)	18 (60%)
Thursday-Friday	17 (23.3%)	10 (23.3%)	7 (23.3%)
Saturday-Sunday	19 (26.0%)	14 (32.6%)	10 (33.3%)
**Time of Day**			
00:01–06:00	6 (8.2%)	4 (9.3%)	2 (6.7%)
06:01–12:00	20 (27.4%)	11 (25.6%)	9 (30.0%)
12:01–18:00	26 (35.6%)	16 (37.2%)	10 (33.3%)
18:01–24:00	21 (28.8%)	12 (28.9%)	9 (30.0%)

Across all MCIs, ambulances were the first arriving rescue vehicles in 79.5% of incidents, followed by Emergency Physician Vehicles (EPVs) (11.0%) And rescue Helicopters (5.5%). Triage was documented before the start of evacuation in 84.9% of cases. For the first evacuated patient, triage category was recorded as “red” in 17.8% and “yellow” in 13.7% of cases, although this information was missing in 65.8% of records. Additional rescue resources were requested in 20.5% of MCIs. These patterns were comparable between the pre- and post-implementation periods.

### Post implementation casualty reporting and system comparison

**Fig. 2:**
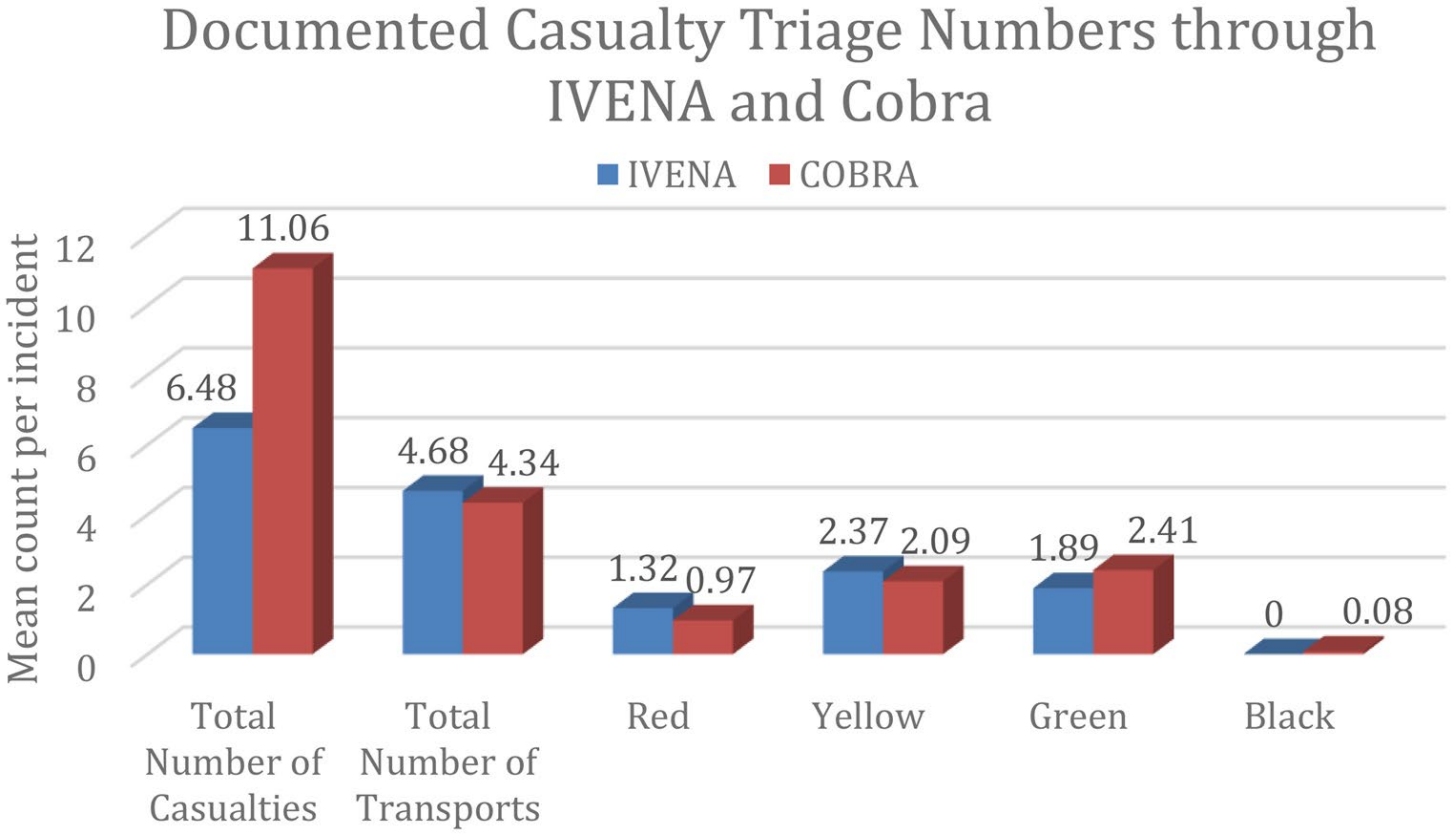
Documented casualty triage numbers (mean) through IVENA and cobra

Analysis of the 30 post-implementation MCIs revealed differences in how casualties were captured and logged between the two platforms. In terms of casualty documentation, COBRA recorded a substantially higher mean number of casualties per incident (11.06 ± 24.02; range 3–200) compared to IVENA (6.48 ± 3.06; range 2–15). (Fig. 2). Despite the difference in total counts, the distribution across triage categories remained broadly consistent between both systems. In both cohorts, Yellow (Urgent) and Green (Minor) patients constituted the majority of documented casualties, while “Black” (Deceased) entries remained near zero (Supplementary Table 1). Complete data synchronization was infrequent. Only 23.3% of incidents showed identical transport counts across both systems. Furthermore, in 17.8% of incidents, the first patient evacuation (COBRA timestamp) began before the first digital distribution log was generated in IVENA.

Individual patient characteristics, including triage, demographics, transport type, diagnosis, and hospital allocation, were available only for the post-implementation cohort (*n* = 188). See Supplementary Data 2 for details.

### Evaluation of prehospital time intervals

**Fig. 3:**
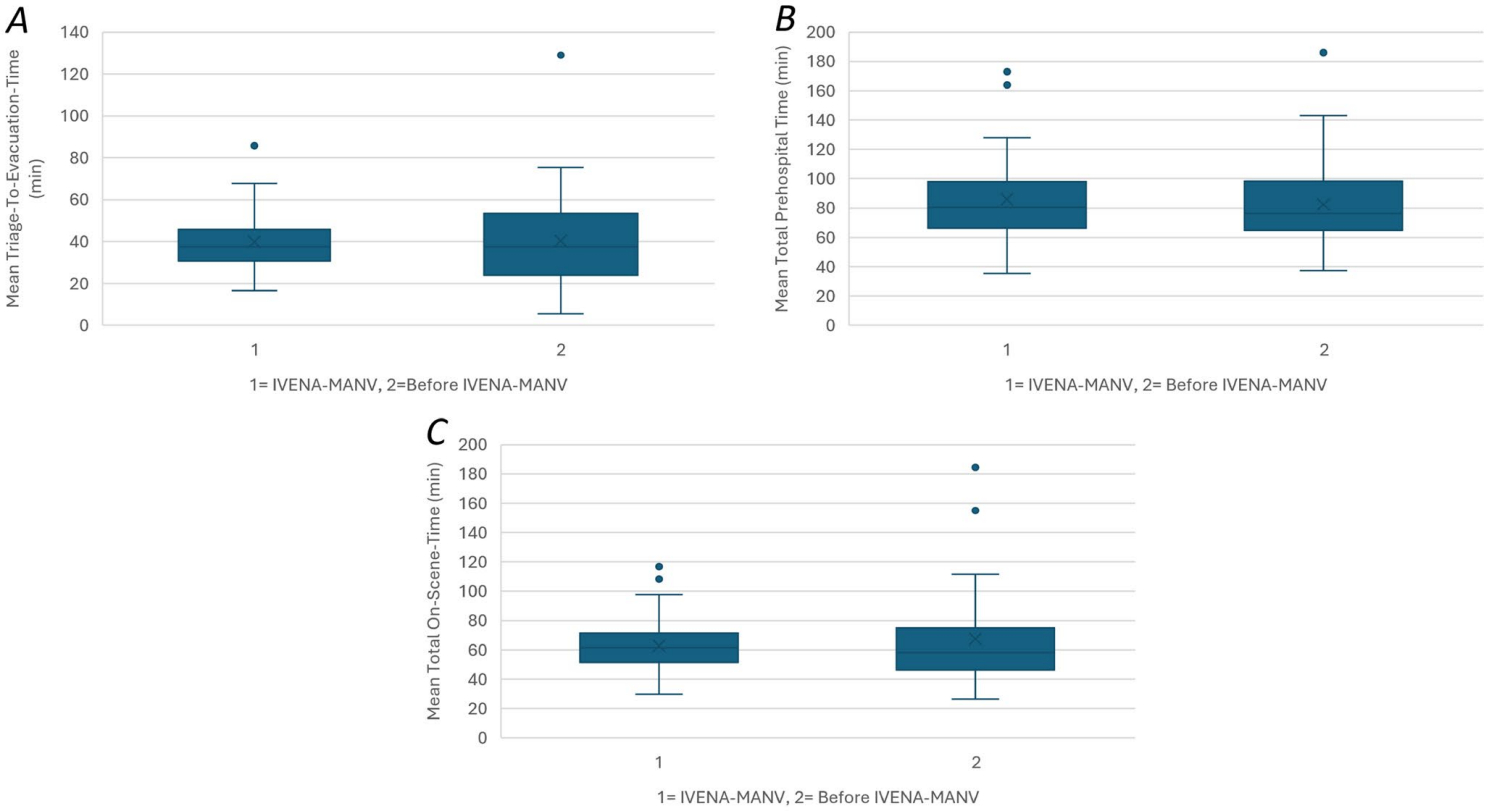
Boxplots with Comparison **A**) of mean Triage-To-Evacuation-Time **B**) Mean Total Prehospital Time and **C**) Total On-Scene Time in minutes before and after the implementation of IVENA-MANV

In the pre-implementation phase, MCI event durations were considerably longer, with a mean of 314.5 ± 245.5 min (range 115.0–1342.0), whereas the post-implementation phase saw a descriptive decrease to 75.7 ± 40.2 min (range 15.0–210.0). Within the post-implementation cohort, the IVENA-MANV system was activated at a mean interval of 19.6 ± 10.5 min following the initial dispatch alarm. Digital coordination processes in this group proved rapid, with initial hospital capacity confirmations received within 1.8 ± 2.2 min and a full capacity overview for the entire incident completed within 29.5 ± 22.5 min. Coordination efficiency was further reflected by a short interval of 8.8 ± 5.8 min between the first digital transport assignment and actual patient evacuation. Despite these streamlined digital processes, analysis of primary operational outcomes, including a mean TtE time of 40.17 ± 19.32 min and a mean TPHT of 83.89 ± 29.53 min, showed no statistically significant differences between the pre- and post-implementation periods (U = 517, *p* = 0.901 for TtE; U = 609.5, *p* = 0.691 for TPHT; U = 642.0, *p* = 0.973 for TOS) (Table 2; Fig. 3). Multiple linear regression analysis confirmed that MCI level was the only significant predictor of prolonged prehospital intervals (*p* < 0.001), while the use of IVENA-MANV did not demonstrate a significant impact on TtE (*p* = 0.244), TPHT (*p* = 0.601), or TOS (*p* = 0.056). Notably, the transition to digital documentation improved data integrity; while seven cases in the pre-implementation group lacked triage timestamps, no missing triage data occurred in the IVENA-MANV cohort, though some entries were recorded with delayed timestamps.

**Table 2:** Comparison times in minutes before and after IVENA MANV implementation

	Mean ± SD (Min-Max)	Mann Whitney-U-Test	Predictor IVENA-MANV	Predictor MCI-Level
TtE Mean	40.17 ± 19.32(5.50–129.0)	517; *p* = 0.901	5.46 ± 4.64; *p* = 0.244	2.04 ± 0.55; *p* = < 0.001
TPHT Mean	83.89 ± 29.53 (35.4–186.0)	609.5; *p* = 0.691	3.16 ± 6.02; *p* = 0.601	3.91 ± 0.70; p = < 0.001
TOST	65.58 ± 27.91 (26.3–184.6)	642.0; *p* = 0.973	11.12 ± 5.71; *p* = 0.056	3.66 ± 0.66; p = < 0.001
TtE First Evacuation	25.02 ± 13.89 (3.0–62.0)	426.0; *p* = 0.664	-2.02 ± 3.93; *p* = 0.600	-0.21 ± 0.44; *p* = 0.643
TtE Last Evacuation	56.42 ± 34.12 (6.0–186.0)	490.5; *p* = 0.639	8.13 ± 8.21; *p* = 0.326	3.57 ± 0.97; p = < 0.001

TtE = Triage-To-Evacuation-Time; TPHT = Total Prehospital Time; TOS = Total On Scene Time. Descriptive Statistics, Mann-Whitney-U-Test (U; p-value) and multivariate regression analysis (regression coefficient B ± standard error; p-value)

To assess individual evacuation efficiency, time intervals were analyzed chronologically for each transport (T1 through T9) within an incident (Table 3). Consistently TtE, TPHT and TOS intervals increased as the sequence of evacuations progressed. Mann-Whitney U tests indicated no statistically significant differences between the pre- and post-implementation cohorts for any individual transport interval. Multiple linear regression models confirmed these findings, with the implementation of IVENA-MANV failing to serve as a significant predictor for reduced time intervals across all metrics, including TtE for the first transport (T1; *p* = 0.603) and the second transport (T2; *p* = 0.191). In contrast, the MCI level was a robust and significant predictor for nearly all individual transport durations (*p* < 0.001 for TPHT T2-T4 and TOS T2-T4).

**Table 3:** Impact of digital coordination on individual patient evacuation intervals before and after IVENA MANV implementation

Transport Sequence	Mean ± SD (Min-Max)	IVENA vs. Pre (*p*-value)^a^	Predictor: IVENA-MANV (β ± SE; *p*)	Predictor: MCI Level (β ± SE; *p*)
**Triage-To-Evacuation Time**				
TtE T1	25.02 ± 13.89 (3.0–62.0)	413.0 ; *p* = 0.647	-2.02 ± 3.87; *p* = 0.603	0.0.21 ± 0.45; *p* = 0.647
TtE T2	34.76 ± 21.18 (7.0–152.0)	409.0 ; *p* = 0.497	7.39 ± 5.58; *p* = 0.191	1.66 ± 0.64; *p* = 0.012
TtE T3	42.40 ± 22.07 (5.0–154.0)	345.5 ; *p* = 0.364	10.41 ± 5.7; *p* = 0.073	2.22 ± 0.65; *p* = 0.001
TtE T4	50.3 ± 24.97 (6.0–157.0)	189.0 ; *p* = 0.146	13.82 ± 6.53; *p* = 0.040	3.08 ± 0.82; p = < 0.001
TtE T5	62.77 ± 36.34 (17.0–186.0)	74.5 ; *p* = 0.678	8.82 ± 14.22; *p* = 0.541	2.73 ± 1.71; *p* = 0.124
TtE T6	63.4 ± 22.09 (36.0–90.0)	4.5 ; *p* = 0.109	20.1 ± 13.22; *p* = 0.172	4.08 ± 4.32; *p* = 0.376
TtE T7	77.83 ± 31.67 (43.0–126.0)	2.0 ; *p* = 0.275	26.33 ± 25–74; *p* = 0.365	-
TtE T8	100.5 ± 47.18 (47.0–162.0)	0.0 ; *p* = 0.121	58.0 ± 40.7; *p* = 0.290	-
TtE T9	80.0 ± 24.04 (63.0–97.0)	-	-	-
**Total Prehospital Time**				
TPHT T1	64.64 ± 22.98 (20.0–130.0)	497.5 ; *p* = 0.316	7.30 ± 5.6; *p* = 0.197	1.27 ± 0.71; *p* = 0.079
TPHT T2	72.85 ± 27.16 (25.0–189.0)	594.0 ; *p* = 0.567	9.31 ± 6.04; *p* = 0.127	2.73 ± 0.70; p = < 0.001
TPHT T3	84.92 ± 31.17 (31.0–221.0)	524.0 ; *p* = 0.974	6.08 ± 7.27; *p* = 0.406	3.11 ± 0.81; p = < 0.001
TPHT T4	90.59 ± 35.49 (42.0–239.0)	263.5 ; *p* = 0.268	6.96 ± 7.81; *p* = 0.377	5.40 ± 0.93; p = < 0.001
TPHT T5	122.17 ± 68.09 (40.0–326.0)	93.0 ; *p* = 0.629	-31.45 ± 24.94; *p* = 0.219	3.93 ± 2.77; *p* = 0.167
TPHT T6	110.9 ± 28.34 (67.0–156.0)	6.0 ; *p* = 0.175	32.01 ± 18.94; *p* = 0.135	-1.67 ± 2.71; *p* = 0.557
TPHT T7	126.0 ± 30.92 (84.0–168.0)	6.0 ; *p* = 1.000	-2.67 ± 30.88; *p* = 0.935	0.37 ± 4.37; *p* = 0.937
TPHT T8	144.20 ± 38.37 (90.0–189.0)	1.0 ; *p* = 0.248	40.0 ± 29.83; *p* = 0.312	3.60 ± 3.65; *p* = 0.428
TPHT T9	156.0 ± 78.35 (91.0–243.0)	0.0 ; *p* = 0.221	-	13.05 ± 3.72; *p* = 0.177
**Total On Scene Time**				
TOS T1	46.75 ± 17.54 (13.0–98.0)	623.0 ; *p* = 0.936	4.02 ± 4.05; *p* = 0.325	1.47 ± 0.47; *p* = 0.002
TOS T2	56.85 ± 24.27 (22.0–178.0)	577.5 ; *p* = 0.597	10.26 ± 5.23; *p* = 0.054	2.85 ± 0.60; p = < 0.001
TOS T3	65.63 ± 25.55 (29.0–180.0)	434.5 ; *p* = 0.331	11.38 ± 5.68; *p* = 0.049	3.08 ± 0.63; p = < 0.001
TOS T4	73.32 ± 26.04 (32.0–183.0)	225.0 ; *p* = 0.091	10.51 ± 6.20; *p* = 0.097	3.36 ± 0.74; p = < 0.001
TOS T5	88.52 ± 39.39 (35.0–227.0)	99.50 ; *p* = 0.843	2.805 ± 14.18; *p* = 0.845	3.24 ± 1.59; *p* = 0.052
TOS T6	89.80 ± 23.57 (55.0–137.0)	3.50 ; *p* = 0.069	25.55 ± 14,51; *p* = 0.122	1.24 ± 4.74; *p* = 0.801
TOS T7	98.0 ± 22.70 (62.0–133.0)	2.0; *p* = 0.157	26.0 ± 18.61; *p* = 0.235	-1.30 ± 2.63; *p* = 0.647
TOS T8	152.33 ± 72.14 (66.0–273.0)	3.0 ; *p* = 0.513	-9.17 ± 83.44; *p* = 0.919	3.75 ± 11.20; *p* = 0.760
TOS T9	188.5 ± 114.49 (82.0–334.0)	0.0 *P* = 0.121	236.0 ± 27.71; *p* = 0.74	-11.0 ± 3.2; *p* = 0.18

TtE = Triage-To-Evacuation-Time; TPHT = Total Prehospital Time; TOS = Total On Scene Time. Descriptive Statistics, Mann-Whitney-U-Test (U; p-value) and multivariate regression analysis (regression coefficient B ± standard error; p-value)

^a^ Mann-Whitney U Test p-value. Multivariate regression coefficients (β ± Standard Error) adjusted for MCI Level and IVENA-MANV implementation

Regarding internal coordination timestamps within the IVENA-MANV cohort (*n* = 24 incidents), the mean interval from initial triage documentation to digital hospital allocation was 28.4 ± 20.3 min (range 3.0–73.0). For the primary transport (T1), this interval was 18.9 ± 16.4 min (range 0.0–50.0). Post-hoc power analysis identified very small effect sizes (d < 0.15) for all primary variables (TtE, TPHT, and TOS), with a corresponding statistical power ranging from 5.1% to 8.0% for the detection of significant differences between the study periods.

### Hospital allocation

To evaluate hospital allocation pattern, the coefficient of variation (CV) for patient distribution per incident was analyzed for events involving multiple hospital types (*n* = 42). The balance of patient distribution did not differ significantly between study periods, with a median CV of 136.9% in both the pre-implementation (IQR 104.6–149.1%) and post-implementation cohorts (IQR 122.5–141.4%; U = 150.5, *p* = 0.777) (Table 4). The number of hospital types utilized per incident also remained consistent; in both periods, approximately half of the incidents utilized two hospital types, while use of three or more types occurred in 43.8% of pre-implementation and 50.0% of post-implementation cases (Table 4).

**Table 4:** Patient distribution balance for the pre and post implementation periods

	Before IVENA-MANV n (*N* = 32)	After IVENA-MANV n (*N* = 10)	*p*-value^a^
**Coefficient of variation (%)**			
Median (IQR)	136.9 (104.6–149.1)	136.9 (122.5–141.4)	*p* = 0.777
Mean ± SD (Range)	133.0 ± 23.8	132.2 ± 19.7	
**Hospital types used per incident**			
2 types, n (%)	18 (56.3%)	5 (50.0%)	
3 types, n (%)	10 (31.3%)	4 (40.0%)	
4 types, n (%)	3 (9.4%)	1 (10.0%)	
5 types, n (%)	1 (3.1%)	0 (0.0%)	

CV = Coefficient of Variation calculated as (SD of patient per hospital/Mean n of patient per hospital) x 100 for patient distribution across hospital types per incident. Analysis limited to incidents with transports to 2 or more hospital types. Available hospitals in the region: Level I trauma centers (*n* = 4), Level II trauma centers (*n* = 8), Level III trauma centers (*n* = 3), non-trauma center (*n* = 1), pediatric hospital (*n* = 1). ^a^Mann-Whitney U test

Hospital allocation appropriateness was assessed for 147 individual transports in the post-implementation period that contained complete triage and allocation data. The overall appropriateness rate was 93.2%. When stratified by triage category, green patients reached appropriate facilities in 100% of cases, followed by yellow patients at 96.7% and red patients at 83.3%. In total, ten high-acuity allocations were classified as inappropriate: eight red patients were transported to Level III trauma centers or non-trauma hospitals, and two yellow patients were transported to non-trauma facilities. All allocations to the available pediatric hospital were considered appropriate, and non-trauma diagnoses (such as intoxications or inhalation injuries) permitted allocation to non-trauma centers regardless of the triage category (Table 5).

**Table 5:** Hospital allocation patterns by triage category based on IVENA-MANV documentation

Triage-Category	Total (*n*)	Level I trauma center	Level II trauma center	Level III trauma center	Pediatric Hospital	Non-trauma-hospital	Appropriate *n* (%)
Red	48	39	1	4	0	4	40 (83.3%)
Yellow	60	41	1	13	3	2	58 (96.7%)
Green	39	16	0	10	6	7	39 (100%)
Total	147	96	2	27	9	13	137 (93.2%)

*N* = 147 with complete triage and hospital allocation data. Appropriateness criteria: Red patients: Level /II trauma centers; Yellow patients: Level I-III trauma centers; Green patients: any hospital type. Pediatric hospital transports were always considered appropriate regardless of triage category. Non-trauma diagnoses (if documented) permitted transport to a non-trauma hospital

## Discussion

This study provides the first real-world evaluation of the digital coordination system IVENA-MANV in MCIs within the Hannover region (Germany). In our findings, the implementation of IVENA-MANV did not significantly influence prehospital time intervals. Instead, the multiple linear regression models identified the incident severity (MCI level) as the dominant predictor of prolonged intervals.

These findings suggest that operational workload and incident complexity, rather than the coordination tool, drive time metrics, aligning with previous simulation [14, 15] and real-world studies [5, 20]. A critical observation in our data was the “documentation lag,” where COBRA-logged evacuations preceded IVENA-MANV entries in 17.8% of cases. This suggests that in high-acuity scenarios, first responders prioritize immediate transport via established radio-based routines over digital registration. This “parallel processing” indicates that digital tools may initially introduce a cognitive or administrative overhead [21]. Also, these aspects underscore challenges in integrating new digital tools into complex emergency workflows and may have blunted potential time-saving effects [22–25]. Several factors may have affected potential time-saving effects during the study period. The first year after the introduction of IVENA-MANV consisted largely of test-runs, followed by only partial implementation followed next year. Consequently, only half of MCI-incidents were documented in both systems. Frequently, transports were being logged before a digital allocation was finalized. Moreover, the binary grouping of pre- versus post-IVENA-MANV fails to capture the nuance of “quality of use”, as key functionalities (including the PZC) were inconsistently applied. These challenges underscore the friction inherent in integrating new digital tools into established, high-stress emergency workflows.

An important methodologic consideration is that the primary outcomes were documented via COBRA radio timestamps that remained unchanged between study periods. Consequently, these intervals may not directly capture IVENA-MANV’s core digital coordination functionality. Rather, any potential effects would be indirect, mediated through operational improvements such as faster hospital allocation decisions are more balanced patient distribution.

Aside from the importance of time as a critical determinant of patient mortality [9–11], one key focus is to ensure the transfer to the correct hospital at the first attempt [20, 26, 27]. Selecting an appropriate trauma center has the potential to reduce mortality of critical casualties [28]. Furthermore, the concept of hospital surge capacity is crucial for MCI management, as both prehospital and in-hospital systems are strained during MCIs [4, 26, 27], and clear command structure and real-time hospital capacity information are essential to prevent overload at the nearest hospitals [27, 28]. IVENA-MANV enabled rapid hospital capacity confirmation with a mean of 1.8 min to first confirmation, and allowed structured patient allocation, linking each patient to specific ambulances and hospitals. This functionality improves documentation, real-time patient tracking, and analysis of secondary transfers or outcomes [29]. The overall appropriateness rate of 93.2% in the post-implementation period demonstrates that IVENA-MANV supported guideline-concordant allocation decisions for most MCI casualties. Focusing on optimal allocation, rather than sheer evacuation speed, reflects a maturing understanding of MCI management as a coordination problem rather than a solely a race [7, 8, 20, 27, 28]. The implementation of a unified digital system also opens possibilities for joint training opportunities between prehospital and hospital teams, which is essential to improve MCI-response [1, 7, 30, 31]. 

Moreover, effective MCI management relies on pragmatism and simplicity with systems that can be used in all types of MCIs by anyone and at any time, especially as systems are constantly changing and evolving [3, 20, 32, 33]. Systems like IVENA-MANV represent a promising path toward optimizing data flow and equitable patient distribution. In the studied region, the dispatch centre maintains parallel capacity for manual distribution, ensuring continuity in case of technical or network failures. This redundancy balances innovation with reliability, a critical consideration in disaster response environments.

This study has several limitations that warrant consideration. Firstly, its retrospective design and reliance on manual radio status updates introduce the potential for delayed or omitted entries. In the pre-implementation group, COBRA radio logs represented the only available data source; in the post-implementation group, the dual documentation provided a parallel, independent source for verification.

Secondly, the study’s sample size resulted in a post-hoc power analysis below 10% for all primary comparisons, suggesting limited ability to detect small effects. To adequately detect small to medium effects substantially larger sample sizes would be required.

Thirdly, the predominance of small-scale MCI in a single dispatch region also limits the generalizability. From a technical standpoint, IVENA-MANV does not impose a fixed upper limit on registered patients, suggesting architectural scalability is not a primary barrier. However, empirical evidence on digital coordination tools at larger scale MCIs with over 100 casualties is lacking. Whether the coordination advantages observed in the smaller incidents are maintained, or whether cognitive overload and infrastructure dependency becoming limiting factors, remains an open question.

Finally, the binary classification of IVENA usage does not fully capture potential variability in system adherence and technical failures. Evidence of COBRA-logged evacuations preceding IVENA-MANV entries suggests incomplete digital workflow integration in some cases, potentially due to operational prioritization.

Despite these limitations, the study provides valuable insights into the qualitative benefits of digital MCI coordination systems in routine operational settings. Future prospective, multicenter studies with larger sample sizes, and mixed-methods approaches are needed to comprehensively evaluate both quantitative and qualitative aspects of digital coordination tools.

## Conclusion

While IVENA-MANV significantly improves documentation and hospital coordination, these digital benefits do not translate into faster scene evacuation. Our findings show that physical incident complexity (and not technology) remains the primary driver of prehospital time intervals. The value of digital tools lies in quality over speed: they ensure guideline-concordant hospital allocation and prevent system-wide overloading. For disaster planners, this study proves that digital platforms should be evaluated on their ability to provide situational awareness and patient safety, rather than as a tool to beat the clock.

## Electronic supplementary material

Below is the link to the electronic supplementary material.

Supplementary Material 1

## Data Availability

The data is available from the corresponding author on reasonable request.
