# Supplementary material for: Digital coordination in mass casualty incidents: a retrospective analysis of prehospital distribution times before and after IVENA-MANV implementation
Source: Eur J Trauma Emerg Surg. 2026 May 20;52(1):168. doi: 10.1007/s00068-026-03216-2 (PMC13190475; doi:10.1007/s00068-026-03216-2)
Supplement: Supplementary file 1 — Supplementary Material 1 [file 68_2026_3216_MOESM1_ESM.docx]

*Supplementary Data 1. Descriptive Statistics for Casualty Characteristics.*

| **Item (Number of)** | **IVENA (Mean ± SD)** | **IVENA (Min-Max)** | **COBRA (Mean ± SD)** | **COBRA (Min-Max)** |
| --- | --- | --- | --- | --- |
| **Total Casualties** | 6.48 ±3.06 | 2–15 | 11.06 ±24.02 | 3–200 |
| Red (SK1) | 1.32 ±1.57 | 0–5 | 0.97 ±1.24 | 0–6 |
| Yellow (SK2) | 2.37 ±1.57 | 0–5 | 2.09 ±2.52 | 0–13 |
| Green (SK3) | 1.89 ±2.58 | 0–10 | 2.41 ±2.94 | 0–13 |
| Black (SK5) | 0 | 0 | 0.08 ±0.32 | 0–2 |
| **Total Transports** | 4.68 ±2.29 | 0–12 | 4.34 ±1.61 | 2–9 |

*Supplementary Data 2. Individual Casualty Characteristics documented through IVENA.*

| Triage-Category | n (% N = 188) |
| --- | --- |
| Red | 57 (30.3 %) |
| Yellow | 83 (44.1 %) |
| Green | 47 (25.0 %) |
| Age-Group |  |
| 0-10 | 9 (4.9 %) |
| 11-18 | 22 (11.7 %) |
| 19-30 | 24 (12.8 %) |
| 31-60 | 34 (18.1 %) |
| 61-90 | 14 (7.5 %) |
| 99 | 9 (4.9 %) |
| Sex |  |
| Male | 33 (17.6 %) |
| Female | 16 (8.5 %) |
| Transportation Vehicle |  |
| Ambulance* | 134 (71.3 %) |
| Ambulance and EPV | 15 (8.0 %) |
| RH | 9 (4.8 %) |
| PTA | 5 (2.7 %) |
| Diagnosis-Groups |  |
| Smoke / Gas Intoxication | 18 (9.6 %) |
| Polytrauma incl Trauma-Resuscitation | 11 (5.8 %) |
| Isolated Trauma | 27 (14.4 %) |
| Hospital Allocation |  |
| Level I Trauma Center | 96 (51.1 %) |
| Level II Trauma Center | 2 (1.1 %) |
| Level III Trauma Center | 27 (14.4 %) |
| Non-Trauma Hospital | 13 (6.9 %) |
| Pediatric Hospital | 10 (5.3 %) |
